# A “one pot” mass spectrometry technique for characterizing solution- and gas-phase photochemical reactions by electrospray mass spectrometry†

**DOI:** 10.1039/d1ra02581c

**Published:** 2021-05-28

**Authors:** Rosaria Cercola, Natalie G. K. Wong, Chris Rhodes, Lorna Olijnyk, Neetisha S. Mistry, Lewis M. Hall, Jacob A. Berenbeim, Jason M. Lynam, Caroline E. H. Dessent

**Affiliations:** Department of Chemistry, University of York Heslington York YO10 5DD UK caroline.dessent@york.ac.uk

## Abstract

The characterization of new photochemical pathways is important to progress the understanding of emerging areas of light-triggered inorganic and organic chemistry. In this context, the development of platforms to perform routine characterization of photochemical reactions remains an important goal for photochemists. Here, we demonstrate a new instrument that can be used to characterise both solution-phase and gas-phase photochemical reactions through electrospray ionisation mass spectrometry (ESI-MS). The gas-phase photochemistry is studied by novel laser-interfaced mass spectrometry (LIMS), where the molecular species of interest is introduced to the gas-phase by ESI, mass-selected and then subjected to laser photodissociation in the ion-trap. On-line solution-phase photochemistry is initiated by LEDs prior to ESI-MS in the same instrument with ESI-MS again being used to monitor photoproducts. Two ruthenium metal carbonyls, [Ru(η^5^-C_5_H_5_)(PPh_3_)_2_CO][PF_6_] and [Ru(η^5^-C_5_H_5_)(dppe)CO][PF_6_] (dppe = 1,2-bis(diphenylphosphino)ethane) are studied using this methodology. We show that the gas-phase photofragmentation pathways observed for the ruthenium complexes *via* LIMS (*i.e.* loss of CO + PPh_3_ ligands from [Ru(η^5^-C_5_H_5_)(PPh_3_)_2_CO]^+^ and loss of just CO from [Ru(η^5^-C_5_H_5_)(dppe)CO]^+^) mirror the solution-phase photochemistry at 3.4 eV. The advantages of performing the gas-phase and solution-phase photochemical characterisations in a single instrument are discussed.

## Abstract 2

The solution and gas-phase dissociative photochemistry of two ruthenium half-sandwich complexes are analysed with electrospray ionisation mass spectrometry in a novel instrument.

## Graphical abstract

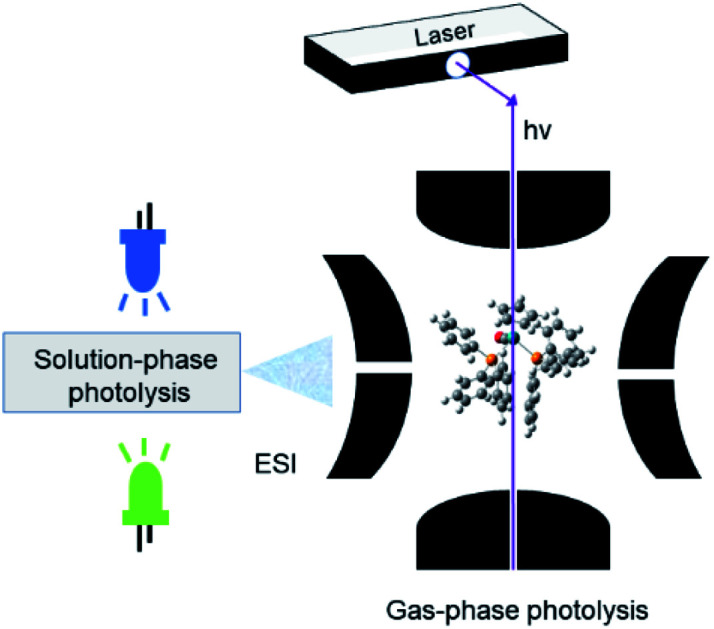

## Introduction

Emerging light-triggered inorganic and organic syntheses drive an increasing need for robust characterization of photochemical transformations.^1–3^ Any reaction intermediates formed during such reactions can exist on dramatically different time scales, and are thus amenable to study by a diverse range of spectroscopic techniques ranging from *in situ* IR through to NMR.^4–6^ Mass spectrometry is an important complementary method to the direct spectroscopic techniques due to its sensitivity which allows identification of very low concentration species. However, its application to photochemical reactions has been limited to date as it is generally an *ex situ* method that requires transfer of a photolyzed solution to the mass spectrometer for analysis.^7,8^ This is despite the fact that it has been widely used to investigate non-photochemical reactions.^9–11^ Very recently, a number of on-line photolysis set ups have been demonstrated that dramatically enhance the potential of mass spectrometry as a tool for monitoring photochemical reactions.^12–14^

In this paper, we describe the application of an instrument that combines on-line photolysis with electrospray ionization mass spectrometry detection and laser-interfaced mass spectrometry (LIMS) to study the photodissociation of CO from metal carbonyl compounds. For the first time, this experiment provides a “one-pot” tool for characterising the solution-phase and gas-phase photochemistry of a system. While the solution-phase measurement provides insight into the real-world photochemistry, the accompanying gas-phase measurement can significantly aid the understanding of the solution-phase mechanism, as well as being directly comparable to high-level quantum chemical calculations. The intrinsic photochemical pathway can also be easier to follow away from the complications of the condensed phase environment. For example, solvation of photochemically generated singlet 16-electron d^6^ metal complexes occurs on a sub-ps timescale so that direct observation of the initial photoproducts in the condensed phase is challenging.^15^

The first report of the use of electrospray ionisation mass spectrometry (ESI-MS) to detect intermediates from a photoinitiated reaction in solution was from Arakawa *et al.*^16^ The study focused on solvolysis of ruthenium complexes, and was able to demonstrate that photoinitiated solvolysis, triggered in the electrospray plume, proceeds by an addition–elimination reaction. Turner *et al.* and co-workers used a similar approach, involving irradiation at the tip of the spray capillary, to study iron cyclopentadienyl complexes catalysing epoxides.^17^ More recently, Badu-Tawiah and co-workers, Chen and co-workers, as well as Roithová and co-workers have employed irradiation in the ESI spray region to characterise the intermediates of photoinitiated catalysis reactions.^12–14^ All of these studies demonstrated that ESI-MS can be used to detect the intermediates of solution-phase photoinitiated reactions, with the caveat that the half-life must be above ∼10 ms. We note that in-source diode light activation was also used by Barran and co-workers to explore the conformational diversity of the UVR8 photoreceptor.^18^

Gas-phase photochemistry experiments that combine photoexcitation and mass spectrometric detection are numerous, although the focus of many of these studies has been on spectroscopy of the mass-selected molecules and clusters rather than the photochemical products.^19^ To provide some specific photochemical examples from this field, Jockusch and Brøndsted Nielsen have studied luminescence of mass-selected ions,^20,21^ and Bieske has employed ion mobility mass spectrometry to study photoswitching reactions.^22^

**Scheme 1:**
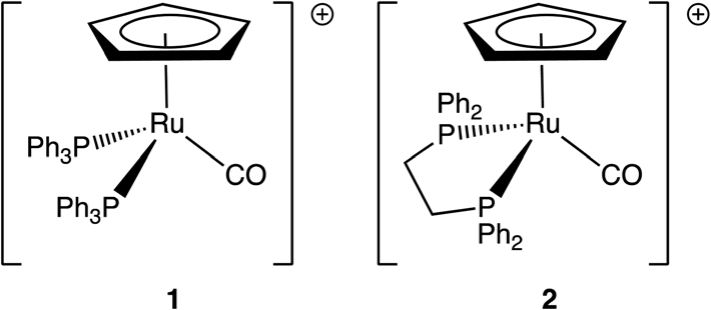
Structures of (1) [Ru(η^5^-C_5_H_5_)(PPh_3_)_2_CO]^+^ and (2) [Ru(η^5^-C_5_H_5_)(dppe)CO]^+^.

We focus here on two ruthenium metal carbonyls, [Ru(η^5^-C_5_H_5_)(PPh_3_)_2_CO][PF_6_] and [Ru(η^5^-C_5_H_5_)(dppe)CO][PF_6_] (dppe = 1,2-bis(diphenylphosphino)ethane) (Scheme 1). Substituted metal carbonyls are widely studied for their photoreactivity, since they are excellent photocatalysts (and precursors) for organic reactions,^23,24^ as well as intermediates for the synthesis of organometallic compounds.^25^ In a number of recent high-profile studies, they were also being investigated for the photocatalytic reduction of CO_2_ to CO or formic acid^26–28^ and to probe the mechanistic pathways that lead to C–C and C–H bond formation.^29,30^ The use of metal carbonyls as CO-releasing molecules (CORMs) is also a promising area of research in medicinal chemistry.^31–37^ Ruthenium half-sandwich complexes have found particular applications in transfer-hydrogenation and alcohol oxidation catalysis,^38–40^ C–H bond functionalisation,^41^ as well as in cancer phototherapy.^42,43^

The CO-releasing properties of metal carbonyl complexes can be tuned to achieve maximum CO photorelease at the required wavelengths by applying the principles of rational design. The metals that are chosen in this study are in a low-spin d^6^ configuration, and can, therefore, access metal-to-ligand charge-transfer transitions (MLCT) leading to M–CO labilisation and CO release. The use of conjugate ancillary ligands, with low-lying π* orbitals, can shift the absorption wavelength to the red, compared to homoleptic metal carbonyls.^44^ However, it is desirable to have robust theoretical methods that can predict such photochemistry, and gas-phase studies are of enormous benefit in this context as they can be readily compared with computational results.^44–46^ In this work, we investigate the intrinsic (*i.e.*, gas-phase) CO releasing photochemistry of two metal carbonyls *via* laser-interfaced mass spectrometry (LIMS),^47,48^ and demonstrate the ability to combine this with online solution-phase photochemistry conducted consecutively in the same instrument. Our novel gas-phase LIMS technique measures all the ionic photoproducts simultaneously with the gaseous absorption spectrum, thus providing a direct measurement of the number of CO units ejected per photon-interaction with the molecule along with the identity of the primary photofragments.^37^

## Experimental

### Chemicals

All chemicals were synthesised according to previously published protocols.^49^

### Laser-interfaced mass spectrometry (gas-phase photochemistry)

Gas-phase UV photodissociation experiments were conducted in a laser-interfaced [Nd:YAG (10 Hz, Surelite) pumped OPO (Horizon)] amaZon ion-trap mass spectrometer (LIMS), which was modified as described in detail elsewhere.^47,50^ The UV spectra were acquired across the range 3.2–5.2 eV (360–238 nm) at ∼1 mJ laser power. A laser step size of 1 nm was employed for all scans. Photodepletion intensity (PD) and photofragment production (PF) were calculated using eqn (1) and (2):1
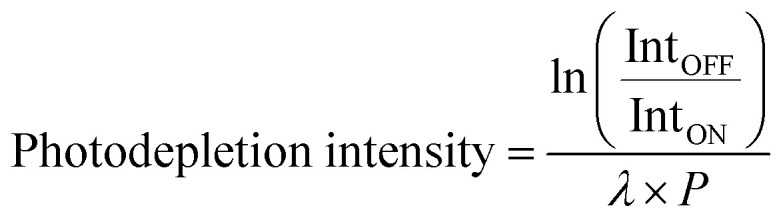
2
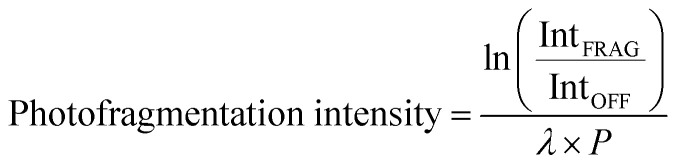
Here, Int_ON_ and Int_OFF_ are the parent ion intensities with laser on and off, Int_FRAG_ the fragment intensity with laser on, *λ* the excitation wavelength (nm) and *P* the laser pulse energy (mJ). The photodepletion spectrum is considered to be equivalent to the gaseous absorption spectrum in the limit where fluorescence is negligible. Quantum ion yields are calculated according to eqn (3):

3 Ion yield = Int_FRAG_/∑Int_PFT_

where Int_PFT_ is the sum of the photofragment ion intensities obtained with the laser on. Higher-energy collisional dissociation (HCD) was performed to investigate the ground-state fragmentation of [Ru(η^5^-C_5_H_5_)(PPh_3_)_2_CO]^+^ and [Ru(η^5^-C_5_H_5_)(dppe)CO]^+^ to complement the LIMS measurements (10^−6^ M solution in DCM : MeOH 3 : 1). An Orbitrap™ Fusion Tribrid mass spectrometer (Thermo Fisher Scientific, Waltham, MA, U.S.A.) was used, as previously described.^51^ HCD breakdown curves were recorded for energies between 0 and 40%. Further details of ESI settings employed are given in Section S2 of the ESI.†

### Online photolysis cell and software

The online photolysis cell was 3D printed (using Autodesk Fusion 360 CAD software and a Makerbot Replicator 2× printer) in the shape of a hollow cuboid (ESI, Section S1, Fig. S1†). On the four long faces LEDs (LuxiGen LZ1 manufactured by LEDEngin, California) of different wavelengths (365, 400, 460 and 523 nm) were mounted in designated cavities. On the two small faces, end caps were printed to access the inside of the cell, which was covered in self-adhesive foil to maximise irradiation from the LEDs (Fig. S1a†). Holes were drilled in the end caps to accommodate a UV transparent fused silica capillary tubing (100 μm ID, 375 μm OD, Molex/Polymicro Technologies, Phoenix, AZ) connected, on one side, to the syringe pump *via* PEEK tubing and on the other, to the ESI needle on top of which the device was mounted (Fig. S1b†). The photolysis cell was controlled with LabVIEW software and an Arduino Nano microcontroller to allow the adjustment of the brightness of one or more LEDs at a time. The individual LED current could be varied from 0 to 1 (where 1 is equal to 700 mA) (Fig. S2†). For the current study, only the 365 nm LED was used, which has a maximum power of 1360 mW with a current of 700 mA.

### Online photolysis experiment

[Ru(η^5^-C_5_H_5_)(PPh_3_)_2_CO][PF_6_] and [Ru(η^5^-C_5_H_5_)(dppe)CO][PF_6_] were electrosprayed at 100 °C from 10^−5^ mol L^−1^ solutions (mixed DCM : MeOH in the ratio 3 : 1) and analysed in positive ion mode. Solution-phase photofragmentation spectra were obtained *via* irradiation of the solutions with the online photolysis cell. The syringe pump flow rate was 0.25 ml h^−1^, and the mass spectra were acquired continuously. A baseline mass spectrum (total ion current) of the solution was acquired for a minute before turning on the LED to provide a background spectrum. Once the LED was turned on, the flow rate was kept constant, allowing irradiation of the solution as it travelled towards the ESI needle.

## Results and discussion

**Fig. 1:**
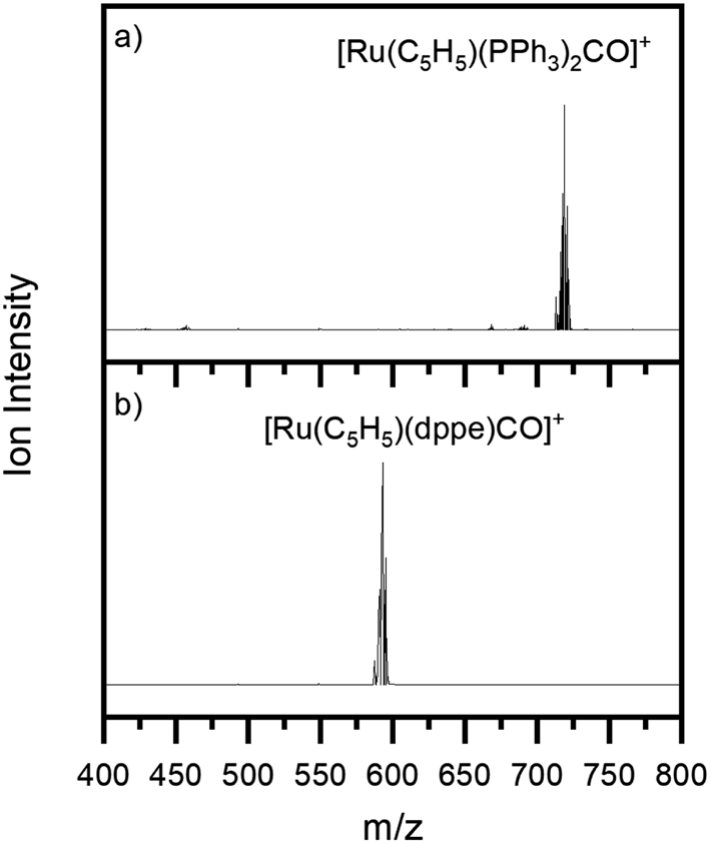
Positive ion mode electrospray mass spectrum of solutions of (a) [Ru(η^5^-C_5_H_5_)(PPh_3_)_2_CO][PF_6_] and (b) [Ru(η^5^-C_5_H_5_)(dppe)CO][PF_6_], illustrating complexes 1 and 2, respectively. The isotopic patterns observed are indicative of complexes containing a single ruthenium atom (Section S3†).

Fig. 1a and b show the ESI-MS of complexes 1 and 2, respectively, run in positive ion mode. We note that the low intensity of fragment ions in these spectra indicates that the parent compounds are representatively transformed from solution to the gas-phase, and do not readily fragment within the source or during the electrospray process.

### Solution and gas-phase absorption spectra of [Ru(η^5^-C_5_H_5_)(PPh_3_)_2_CO]^+^ and [Ru(η^5^-C_5_H_5_)(dppe)CO]^+^

**Fig. 2:**
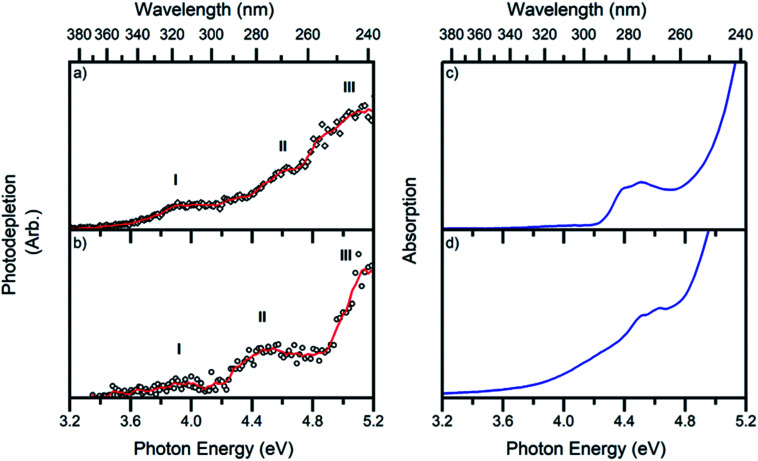
Photodepletion (gas-phase absorption) spectra of complexes (a) 1 and (b) 2. Spectra were recorded across the range 3.2–5.2 eV. The solid lines are five-point adjacent averages of the data points. Solution-phase absorption spectra of (c) [Ru(η^5^-C_5_H_5_)(PPh_3_)_2_CO][PF_6_] and (d) [Ru(η^5^-C_5_H_5_)(dppe)CO][PF_6_] in DCM : MeOH (3 : 1) between 3.2–5.2 eV.

The gas-phase absorption spectra of [Ru(η^5^-C_5_H_5_)(PPh_3_)_2_CO]^+^ and [Ru(η^5^-C_5_H_5_)(dppe)CO]^+^ obtained *via* photodepletion of the mass-selected precursor ions (Fig. 1) are presented in Fig. 2a and b. We note that the gas-phase photolysis experiments were conducted on the cationic chromophores, 1 and 2, whereas the anionic PF_6_^−^ counterions were also present in solution. The presence, or absence, of these counterions will not affect the absorption spectra.

Both 1 and 2 have very similar absorption spectra, and display three distinct bands which are labelled I, II and III peaking at ∼3.9, 4.6 and 5.1 eV for 1 and ∼4.0, 4.5 and 5.2 eV for 2. Band II for complex 1 (Fig. 2a) is more clearly visible in the photofragmentation action spectrum of this species (Fig. 5a). Fig. 2c and d display the solution-phase absorption spectra of [Ru(η^5^-C_5_H_5_)(PPh_3_)_2_CO][PF_6_] and [Ru(η^5^-C_5_H_5_)(dppe)CO][PF_6_], which are in good agreement with the gas-phase absorption spectra. Several points are of note when comparing the gas- and solution-phase spectra: Firstly, the similar spectral profiles observed for the compounds in the gas-phase and solution demonstrates that the gas-phase spectra were obtained *via* single-photon photodissociation. Secondly, the fact that no significant solution-induced spectral shift occurs for these compounds means that gaseous and solution excitation energies accessed the same electronic transitions (Section S4† presents time dependent density functional calculations for 1). Finally, the fact that both spectra are similar confirms that the mass-selected precursor gaseous ion is the major chemical species in the solution-phase, and hence the dominant species that was photolyzed in solution.

### Photodissociation pathways of [Ru(η^5^-C_5_H_5_)(PPh_3_)_2_CO]^+^ and [Ru(η^5^-C_5_H_5_)(dppe)CO]^+^

**Fig. 3:**
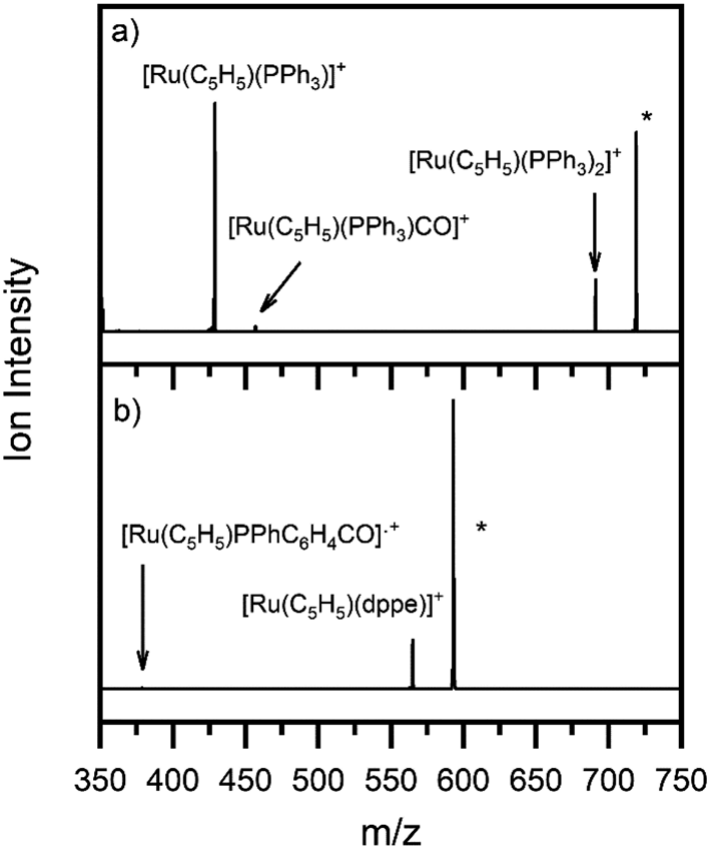
Photofragment mass spectra of complexes (a) 1 and (b) 2 at 4.6 eV. * indicates the precursor ion.

Fig. 3 shows the photofragment mass spectra observed following laser photoexcitation of complexes 1 and 2 at 4.6 eV (270 nm) within the band II region. The main photofragment obtained for complex 1 (Fig. 3a) corresponds to the loss of both a CO and a PPh_3_ ligand from the precursor ion:

4a [Ru(η^5^-C_5_H_5_)(PPh_3_)_2_CO]^+^ + *hν* → [Ru(η^5^-C_5_H_5_)(PPh_3_)]^+^ + CO + PPh_3_

with additional photofragments being produced corresponding to the photoinduced loss of either CO or PPh_3:_

4b [Ru(η^5^-C_5_H_5_)(PPh_3_)_2_CO]^+^ + *hν* → [Ru(η^5^-C_5_H_5_)(PPh_3_)_2_]^+^ + CO

4c [Ru(η^5^-C_5_H_5_)(PPh_3_)_2_CO]^+^ + *hν* → [Ru(η^5^-C_5_H_5_)(PPh_3_)CO]^+^ + PPh_3_

Ejection of PPh_3_ is undesirable for applications aimed at CO photorelease, including ligand substitution.^52^ Compound 2 offers a good alternative in this context to compound 1 since dppe is a chelating ligand and is, therefore, less likely to dissociate from the metallic centre. Photoexcitation of 2 at 4.6 eV produced the photofragment mass spectrum shown in Fig. 3b, with the main photofragment corresponding to the loss of the carbon monoxide unit from the parent ion and the dppe ligand remaining bound to the metal centre:

5a [Ru(η^5^-C_5_H_5_)(dppe)CO]^+^ + *hν* → [Ru(η^5^-C_5_H_5_)(dppe)]^+^ + CO

Indeed, [Ru(η^5^-C_5_H_5_)(dppe)]^+^ was the most significant photofragment observed across the entire spectral range. [Ru(η^5^-C_5_H_5_)(PPhC_6_H_4_)CO]˙^+^ was produced as a minor photofragment by a bond breaking in the dppe ligand (5b):5b
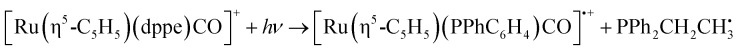


**Fig. 4:**
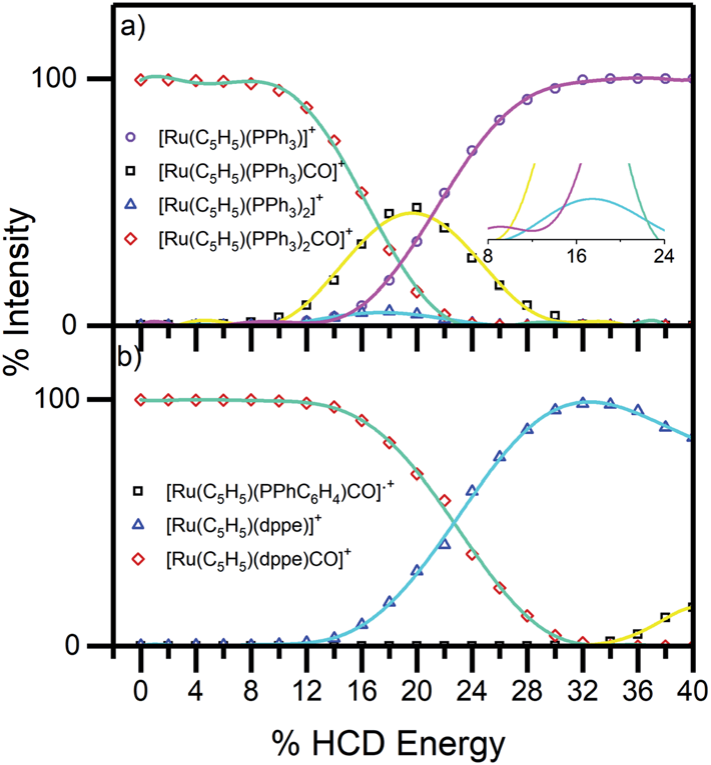
HCD breakdown curves of complex ions (a) 1 and (b) 2 between 0–40% HCD energy, shown with production curves of the resulting fragment ions. The inset in (a) shows the expanded section of the HCD curves between 0–25% and illustrates PPh_3_ and CO loss as in eqn (4b) and (4c).

**Fig. 5:**
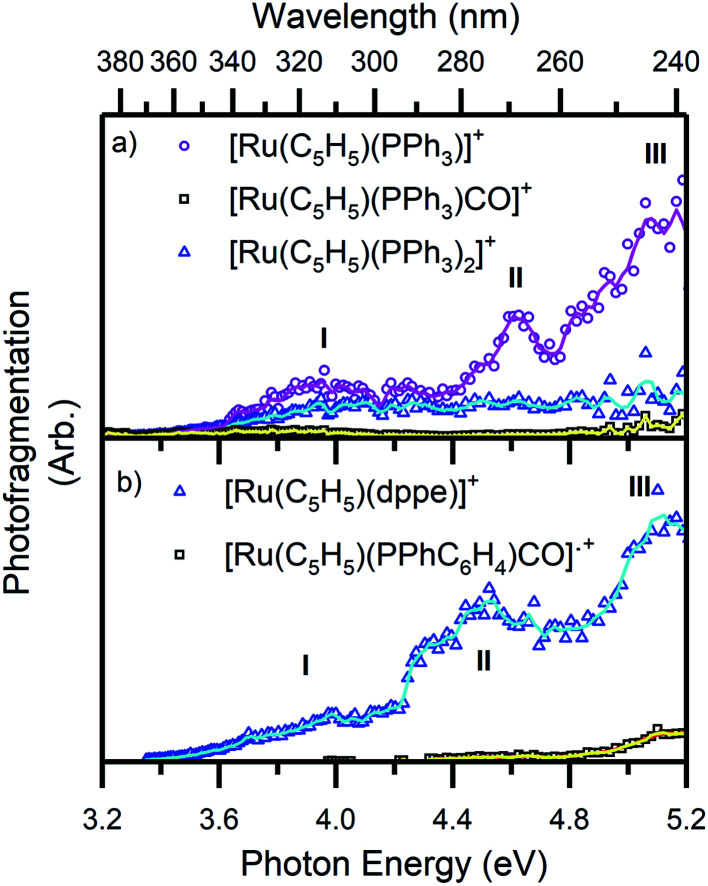
Photofragment production spectra from the complex ions (a) 1 and (b) 2. Spectra are recorded across the range 3.2–5.2 eV. The solid lines are two-point adjacent averages of the data points.

To aid the interpretation of the photofragmentation pathways, it was useful to perform higher-energy collisional dissociation.^51^ This experiment maps out the ground-state fragmentation pathways as a function of internal energy, and can therefore provide insight into the formation pathways of the photofragments. The HCD curves (0–40% collision energy) obtained for complexes 1 and 2 are shown in Fig. 4. Both complexes can be seen to be stable in the gas phase, as neither fragments below 10% HCD energy. Loss of the single CO from compound 1, (4b), represented only a minor pathway between 10–25% HCD energy. In contrast, loss of a single PPh_3_ unit, (4c), is the dominant lower-energy fragmentation channel. These results mirror those of Crawford *et al.* on the [Ru_6_C(CO)_16_(PPh_3_) + OMe]^−^ system,^53^ where the phosphine ligand was also ejected first when the cluster was subjected to collision-induced dissociation. At higher HCD energies (>20%), the [Ru(η^5^-C_5_H_5_)(PPh_3_)]^+^ ion can be seen to be produced as a secondary fragment concomitant with the reduction in [Ru(η^5^-C_5_H_5_)(PPh_3_)CO]^+^. It is notable that [Ru(η^5^-C_5_H_5_)(PPh_3_)]^+^ is the main photoproduct above 3.4 eV (Fig. 5a), suggesting that it may be produced though photoexcitation initially producing hot [Ru(η^5^-C_5_H_5_)(PPh_3_)CO]^+^ that subsequently fragments.

For complex 2, the only significant fragment produced over the HCD range studied corresponded to loss of the single CO ligand and production of [Ru(η^5^-C_5_H_5_)(dppe)]^+^(5a). This ion is also the only significant intensity photofragment. At high energies (>32%), the [Ru(η^5^-C_5_H_5_)(PPhC_6_H_4_)CO]˙^+^ ion is observed (5b) as a secondary fragment.

Fig. 5 presents a wavelength-dependent picture of the photodissociation pathways of complexes 1 and 2, obtained by displaying the photofragment production mass spectra across the full spectral range of the gas-phase absorption spectrum (Fig. 2). Photofragmentation of [Ru(η^5^-C_5_H_5_)(PPh_3_)_2_CO]^+^ (Fig. 5a) can be seen to follow pathways (4a–4c). The most intense photofragment at all energies was [Ru(η^5^-C_5_H_5_)(PPh_3_)]^+^, (4a), produced *via* loss of CO and PPh_3_. In contrast, fragmentation of 1 with loss of the CO moiety, (4b), displays a rather flat profile between 3.8–5.2 eV. Loss of a single PPh_3_ ligand (4c) is a minor channel that shows a modest increase in intensity at the highest energies.

Fig. 5b displays the photofragment production spectra from complex 2, revealing that the [Ru(η^5^-C_5_H_5_)(dppe)]^+^ photofragment, (5a), was the dominant product ion across the spectral range, with a profile that closely matches the gaseous absorption spectrum. The only other observed photofragment, [Ru(η^5^-C_5_H_5_)(PPhC_6_H_4_)CO]˙^+^, (5b), was produced in low quantities from ∼4.3 eV with a small increase between 4.8–5.2 eV, at high ion internal energies.

**Fig. 6:**
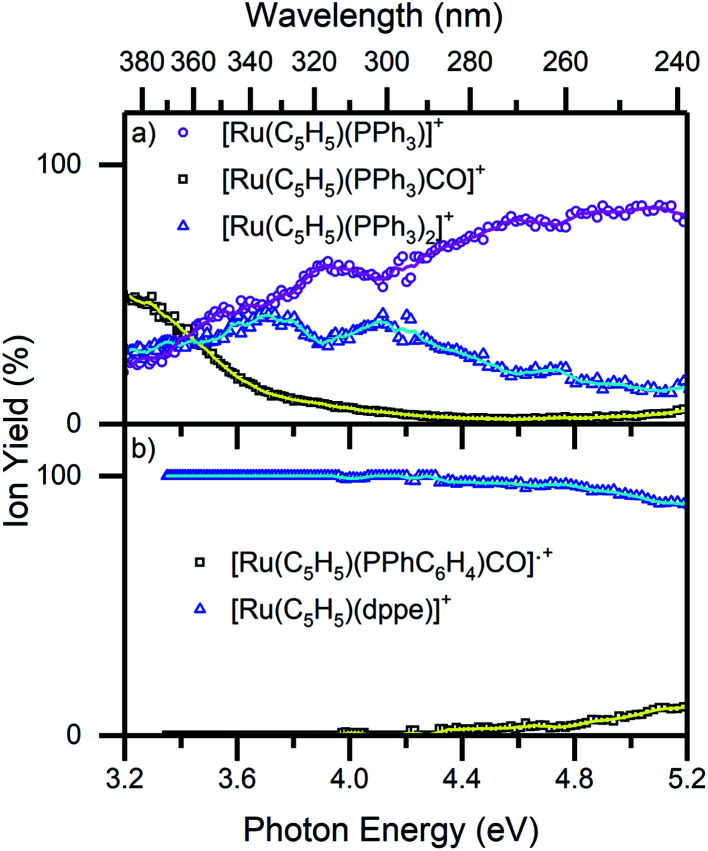
Ion yield spectra of the photofragments produced from (a) 1 and (b) 2 in the region between 3.2–5.2 eV. The solid lines are five-point adjacent averages of the data points.

Fig. 6 displays the photofragmentation data as ion-yield spectra, presenting a clearer picture of the branching into the different photodissociation channels.^37,54^ For 1 (Fig. 6a), the loss of CO and PPh_3_(4a) is the strongest channel at all energies above 3.4 eV, but the loss of just CO (4b) can be seen to be enhanced at energies between 3.7–4.2 eV. At the lowest photoexcitation energies (3.2–3.4 eV), the loss of a single PPh_3_ ligand represents the strongest photofragmentation pathway (4c), although this channel falls away dramatically as the excitation energy, and hence, the internal energy increases. The ion yield curves for complex 2, show that the [Ru(η^5^-C_5_H_5_)(dppe)]^+^ photofragment (5a), is produced with 100% yield up to 4.4 eV, decreasing to ∼90% ion yield at 5.2 eV, due to branching into [Ru(η^5^-C_5_H_5_)(PPhC_6_H_4_)CO]˙^+^(5b).

### Solution-phase photolysis

**Fig. 7:**
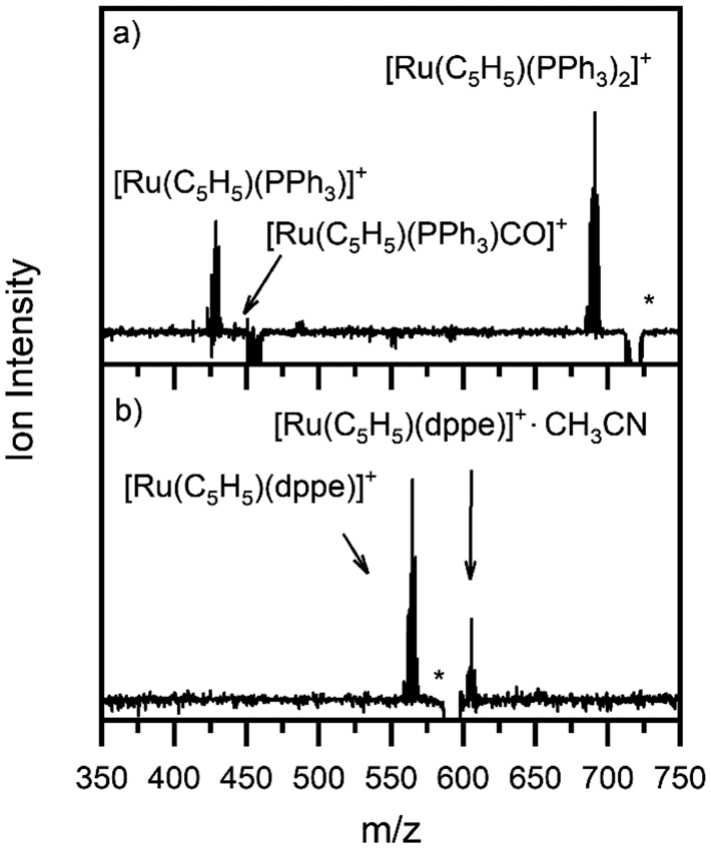
Photolysis_on_–photolysis_off_ off mass spectra of a solution of (a) [Ru(η^5^-C_5_H_5_)(PPh_3_)_2_CO][PF_6_] and (b) [Ru(η^5^-C_5_H_5_)(dppe)CO][PF_6_] after irradiation at 3.4 eV. * indicates the parent ion. [Ru(C_2_H_5_)(PPh_3_)_2_]^+^ is twice the intensity of [Ru(C_2_H_5_)(PPh_3_)]^+^, and ∼20 times that of [Ru(C_2_H_5_)(PPh_3_)CO]^+^. [Ru(C_2_H_5_)(dppe)]^+^ intensity is 2.7 times [Ru(C_2_H_5_)(dppe)·CH_3_CN]^+^.

Fig. 7 shows the photolysis_on_–photolysis_off_ ESI-MS obtained for photoirradiation at 365 nm of 1 and 2 with the photolysis cell. For 1 (Fig. 7a), solution-phase irradiation resulted in the same two main photofragments as those observed upon gas-phase irradiation, *i.e.*, pathways (4a), and (4b), which correspond to the loss of CO, and CO + PPh_3_ units, respectively. The other fragment, [Ru(η^5^-C_5_H_5_)(PPh_3_)CO]^+^, produced *via* the loss of PPh_3_ from 1 (eqn (4c)), was present in the electrosprayed solution (Fig. 1a) and was photolyzed during our experiment, resulting in a negative peak.

The corresponding photolysis_on_–photolysis_off_ mass spectrum of 2 is displayed in Fig. 7b. Upon gas-phase photoexcitation, the dominant photoproduct for this compound corresponded to ejection of CO, and the compound can also be seen to produce the same fragment as the major photoproduct following solution-phase irradiation. A second significant intensity photoproduct is observed for this compound, which is assigned as [Ru(η^5^-C_5_H_5_)(dppe)(CH_3_CN)]^+^, formed *via* reaction of the direct photoproduct [Ru(η^5^-C_5_H_5_)(dppe)]^+^ with acetonitrile traces present in the mass spectrometer inlet or trap. Similar solvent addition products were observed in online photolysis experiments performed by Arakawa *et al.* on bisphenanthroline complex [Ru(phen)_2_B]^2+^ (where phen = 1,10-phenanthroline, B = ethylenediamine, trimethylenediamine, or butanediamine).^16^ We note that the acetonitrile adduct is not observed with [Ru(η^5^-C_5_H_5_)(PPh_3_)]^+^. From the HCD curves (Fig. 4), it is evident that 1 and 2 display different CO binding energies, and the same would be true for an acetonitrile ligand that replaced a CO. If acetonitrile binds more weakly to [Ru(η^5^-C_5_H_5_)(PPh_3_)]^+^ than [Ru(η^5^-C_5_H_5_)(dppe)]^+^, the resulting complex ion might be subject to metastable decay upon electrospray or transit through the mass spectrometer.

## Concluding remarks

This work has demonstrated the use of an ESI-mass spectrometry instrument to probe the gas-phase and on-line solution-phase dissociative photochemistry of two ruthenium half–sandwich complexes, [Ru(η^5^-C_5_H_5_)(PPh_3_)_2_CO]^+^ and [Ru(η^5^-C_5_H_5_)(dppe)CO]^+^. Each compound was found to follow the same primary photofragmentation pathway *i.e.*, loss of CO + PPh_3_ ligands from [Ru(η^5^-C_5_H_5_)(PPh_3_)_2_CO]^+^ and loss of just CO from [Ru(η^5^-C_5_H_5_)(dppe)CO]^+^, both in solution and in the gas phase.

It is useful to discuss the potential benefits of using a single instrument to obtain consecutive gaseous and on-line solution-phase photolysis measurements. First, one clear benefit of conducting solution-phase photolysis to complement the gas-phase measurement, lies in being able to test the relevance of the gaseous results to the more widely-encountered solution-phase environment, where the majority of photochemistry occurs. For systems such as the compounds studied here where the solution and gas-phase photoproducts are the same, the gas-phase production profiles are likely to map those in solution, allowing the single-wavelength diode measurement to be extrapolated to other photoexcitation energies.

From the opposite perspective, what is the benefit of the gas-phase measurement in addition to on-line photolysis? Since the gas-phase measurement is effectively a mass-selective spectroscopic measurement, the precursor species that produces the measured photoproducts is unambiguous. This provides clarity around a number of issues that can complicate solution-phase measurements, including the effect of charge state and aggregation. An additional benefit of performing photodissociation in the gas phase is that the measurement is effectively background free, allowing the detection of very low yield photoproducts. Finally, as the gaseous measurements are performed in the absence of solvent, the direct photoproducts can be identified, with clarity that secondary solvent reactions, or reactions of an excited state molecule with a second precursor molecule are not involved in their formation. This third point illustrates the important synergy for photochemical mechanistic studies which results from doing both gas-phase and solution-phase measurements consecutively, since comparison of the two sets of results allows delineation of mechanisms of photoproduct formation where solvent is involved from those where it is not.^14^

In summary, by linking an on-line photolysis source with a laser-interfaced mass spectrometer, we have demonstrated an instrument that can be used to consecutively characterise photochemical mechanisms in solution and gas phase. The dissociative photochemistry of a pair of CO-releasing ruthenium half-sandwich complexes was characterised using this setup. On-line diode photolysis provides an efficient and rapid tool for photoreaction screening, while the laser interfaced mass spectrometry measurements provide insight into the wavelength-dependent photochemistry across a broad excitation range. This approach could be widely applied to photochemical processes of emerging interest, such as light-activated prodrugs,^55,56^ photocatalysts,^12–14^ and environmental pollutants.^57,58^

## Conflicts of interest

There are no conflicts of interest to declare.

## Supplementary Material

RA-011-D1RA02581C-s001
