# Supplementary material for: A “one pot” mass spectrometry technique for characterizing solution- and gas-phase photochemical reactions by electrospray mass spectrometry
Source: RSC Adv. 2021 May 28;11(32):19500–7. doi: 10.1039/d1ra02581c (PMC9033567; doi:10.1039/d1ra02581c)
Supplement: RA-011-D1RA02581C-s001 [file RA-011-D1RA02581C-s001.pdf]

Electronic Supplementary Material (ESI) for RSC Advances.
This journal is © The Royal Society of Chemistry 2021

Supplementary Information

A "one pot" mass spectrometry technique for characterizing solution- and gas-phase photochemical reactions by electrospray mass spectrometry

Rosaria Cercola, Natalie G. K. Wong, Chris Rhodes, Lorna Olijnyk, Neetisha Mistry, Lewis M. Hall, Jacob A. Berenbeim, Jason M. Lynam and Caroline E. H Dessent*

Department of Chemistry, University of York, Heslington, York YO10 5DD, U.K.

Corresponding author: *caroline.dessent@york.ac.uk

Section S1: On-line photolysis apparatus

Section S2: Additional experimental details

Section S3: Isotope patterns

Section S2: TDDFT calculations of $[Ru(\eta^5\text{-}C_5H_5)(PPh_3)_2CO]^+$

Section S1: On-line photolysis apparatus

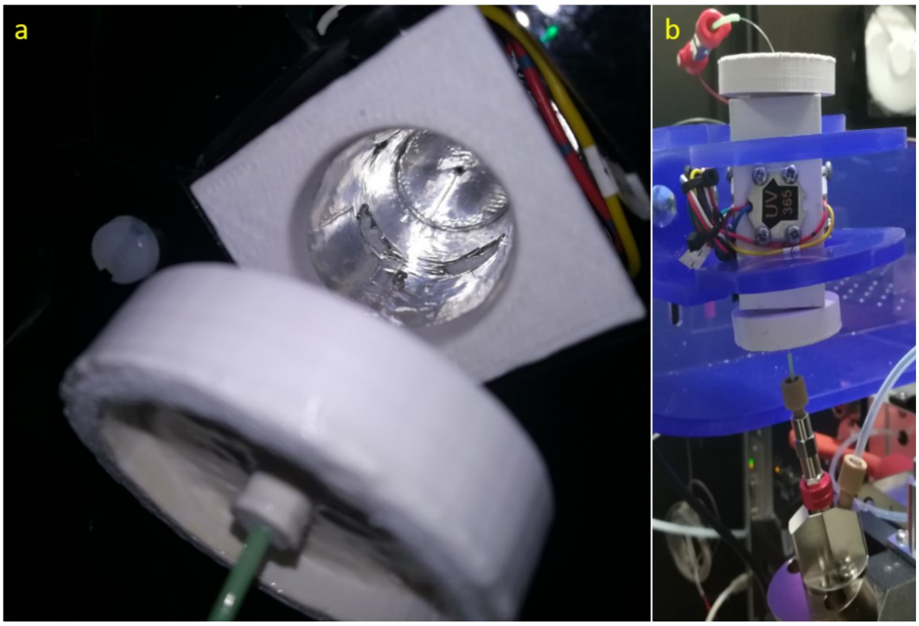

Figure S1: a) Pictures of the inside of the photolysis cell and b) photolysis cell mounted on top of the electrospray needle, in the experimental configuration.

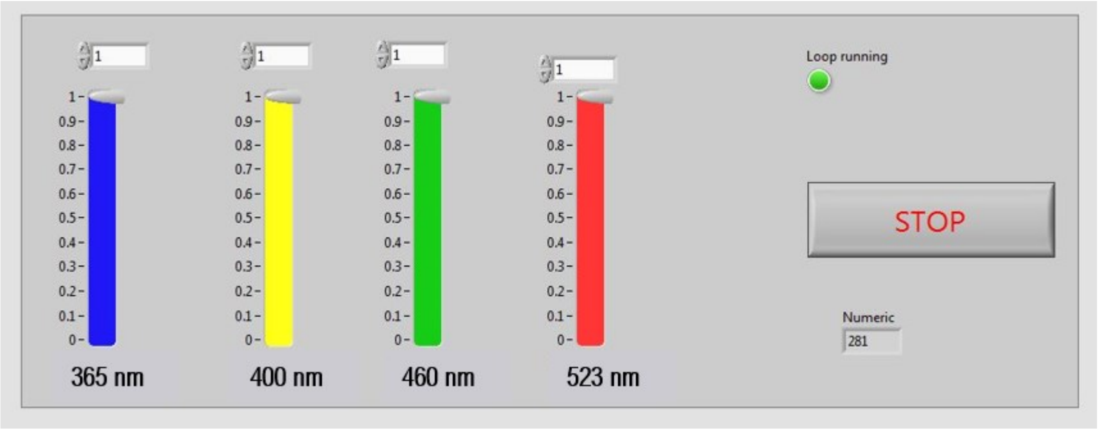

Figure S2: Interface of the LabVIEW software employed with the photolysis cell.

Section S2: Additional experimental details

The ESI voltages used in the experiments conducted were as follows:

Capillary exit block: -200 V: Capillary exit: 140 V: HV Capillary: -3000 V: HV endplate offset: -500 V

Ion guide:

RF Level	99 %
Funnel 1 RF (Pass)	361.13 Vpp
Funnel 1 Entrance (Pass)	100.00 V
Funnel 1 Exit (Pass)	35.00 V
Funnel 1 Lens 1 (Pass)	25.00 V
Funnel 2 RF (Pass)	522.25 Vpp
Funnel 2 Entrance (Pass)	12.00 V
Funnel 2 Exit (Pass)	10.00 V
Funnel 2 Lens 1 (Pass)	2.83 V
Octopole RF (Pass)	142.25 Vpp
Octopole 1 DC (Pass)	2.30 V
Octopole Partition (Pass)	1.70 V
Octopole 2 DC (Pass)	1.10 V
Octopole Delta DC (Pass)	0.00 V
Focus 1 Lens 1 (Pass)	0.50 V
Focus 1 Lens 2 (Pass)	-20.00 V
Focus 1 Lens 3 (Pass)	-6.00 V
Multipole Lens 1 (Pass)	-6.00 V
Multipole RF (Pass)	57.25 Vpp
Multipole DC (Pass)	-4.00 V
Focus 4 Lens 1 (Pass)	-4.00 V
Focus 4 Lens 2 (Pass)	-5.00 V
Focus 4 Lens 3 (Pass)	-75.00 V
Funnel 1 RF (Block)	361.13 Vpp

Funnel 1 RF (Block)	361.13 Vpp
Funnel 1 Entrance (Block)	100.00 V
Funnel 1 Exit (Block)	35.00 V
Funnel 1 Lens 1 (Block)	25.00 V
Funnel 2 RF (Block)	522.25 V
Funnel 2 Entrance (Block)	12.00 V
Funnel 2 Exit (Block)	10.00 V
Funnel 2 Lens 1 (Block)	2.83 V
Octopole RF (Block)	142.25 Vpp
Octopole 1 DC (Block)	2.30 V
Octopole Partition (Block)	1.70 V
Octopole 2 DC (Block)	1.10 V
Octopole Delta DC (Block)	0.00 V
Focus 1 Lens 1 (Block)	0.50 V
Focus 1 Lens 2 Delta (Block)	100.00 V
Focus 1 Lens 3 (Block)	-6.00 V
Multipole Lens 1 (Block)	-6.00 V
Multipole RF (Block)	57.25 Vpp
Multipole DC (Block)	-4.00 V
Focus 4 Lens 1 (Block)	-4.00 V
Focus 4 Lens 2 (Block)	-5.00 V
Focus 4 Lens 3 Delta (Block)	100.00 V

Section S3: Isotope patterns

Figure S2 displays the experimental and simulated isotope pattern for ([Ru(η^5-C_5H_5)(PPh_3)$_2$CO]$^+$). The simulation confirms the assigned identity of the complex ion.

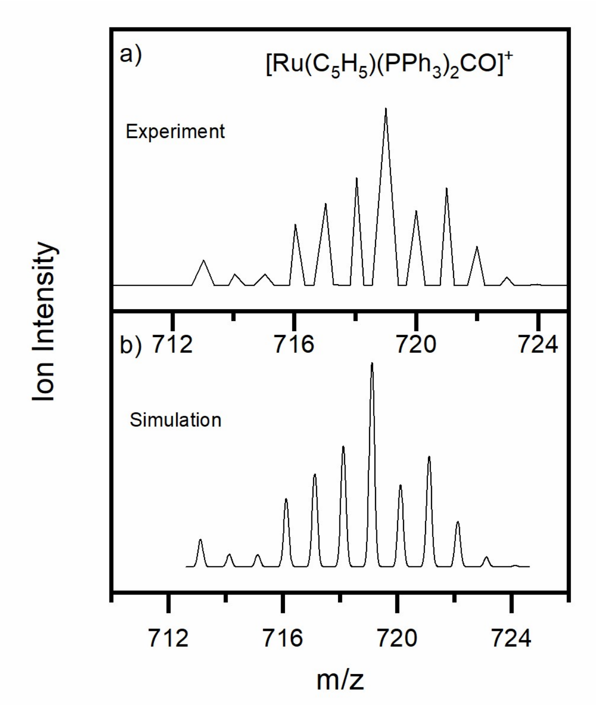

Figure S3 displays the experimental and simulated isotope pattern for ([Ru(η^5-C_5H_5)(dppe)CO]$^+$). The simulation confirms the assigned identity of the complex ion.

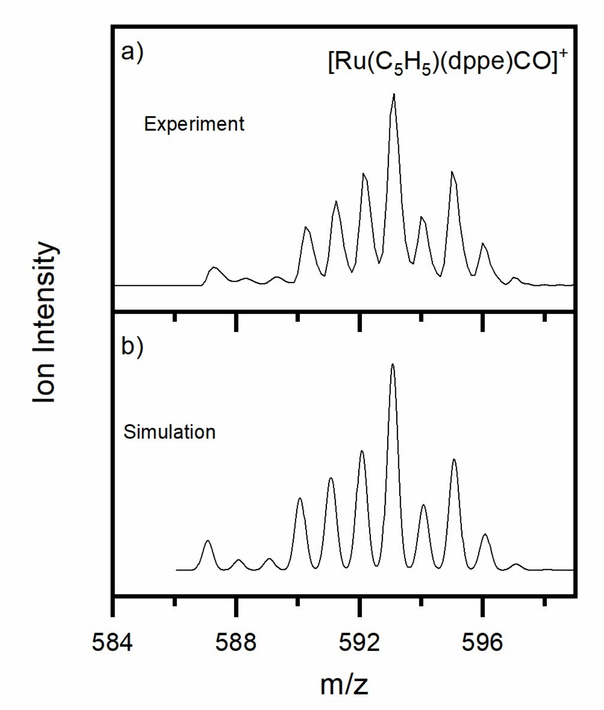

Section S2: TDDFT calculations of [Ru(η^5-C_5H_5)(PPh_3)$_2$CO]$^+$

TDDFT calculations were performed for compound **1 (**[Ru(η^5-C_5H_5)(PPh_3)$_2$CO]$^+$**)** at a number of different levels of theory. Prior to performing the TDDFT calculations, compound **1** was fully optimised at the PBE0/Def2-SV level, with ECP on Ru, with the global minimum structure being displayed in Fig. S3.

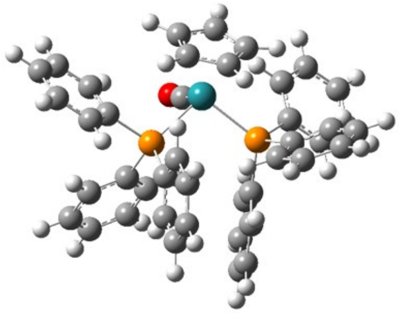

Figure S3: Global minimum structure of compound1 at the PBE0/Def2-SV level with ECP on Ru.

The calculated absorption spectra obtained from the TDDFT calculations are shown in Fig. S4. The B3LYP/6-31G* with SDD on Ru calculated spectrum is closest to the experimental photodepletion spectrum. Table S1 reports a comparison between the experimental and computational excitation energies, and Table S2 lists the main transitions between 3 and 4.7 eV. The orbitals that are involved in these transitions are shown in Fig. S5. The orbital character in these transitions are all a combination of the metal d orbitals and the ligand π orbitals. In all of the density functional calculations studied here, numerous highly-mixed transitions are predicted at energies higher than 4.7 eV.

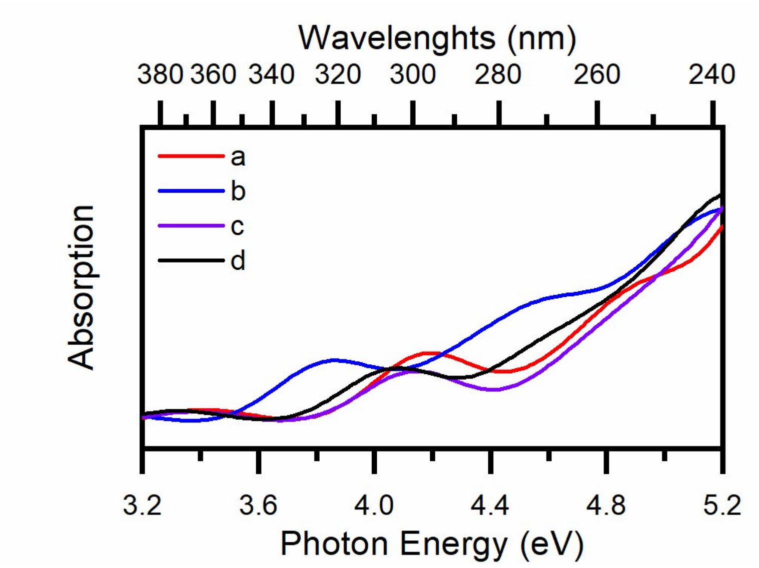

Figure S4: Calculated absorption spectra of complex 1 at a) PBE0/6-31G*, b) B3LYP/6-31G*, c) PBE0/Def2-SV and d) B3LYP/Def2-SV theory levels. ECP core potential of Ru is always included.

Table S1: Comparison between experimental absorption maxima and calculated absorption maxima at different levels of theory. The energies are given in eV.

**Experimental**	**PBE0 6-31G***	**B3LYP 6-31G***	**PBE0 Def2-SV**	**B3LYP Def2-SV**
-	3.4	3.1	3.4	3.3
3.9	4.2	3.8	4.1	4.1
4.6	4.8	4.6	4.8	4.8
5.1	5.4	5.2	5.4	5.2

Table S2: Calculated TDDFT transition energies and oscillator strengths of complex 1. Calculations were performed at the B3LYP/6-31G*/SDD level. Only transitions below 4.7 eV, and with oscillator strength > 0.01 are listed.

**Orbital transitions**	**Photon energy**/eV	**f**
(0.87)170→171	3.06	0.0151
(0.72)170→172	3.75	0.0147
(0.48)169→171<br>(0.10)170→172	3.85	0.0615
(0.54)169→172	4.17	0.0267
(0.52)168→172	4.31	0.0274
(0.25)167→171<br>(0.16)156→172	4.37	0.0199
(0.34)166→171<br>(0.17)168→171	4.51	0.0204
(0.23)166→171	4.57	0.0264
(0.65)170→173	4.60	0.0225
(0.58)162→171	4.67	0.0124
(0.29)170→174	4.67	0.142

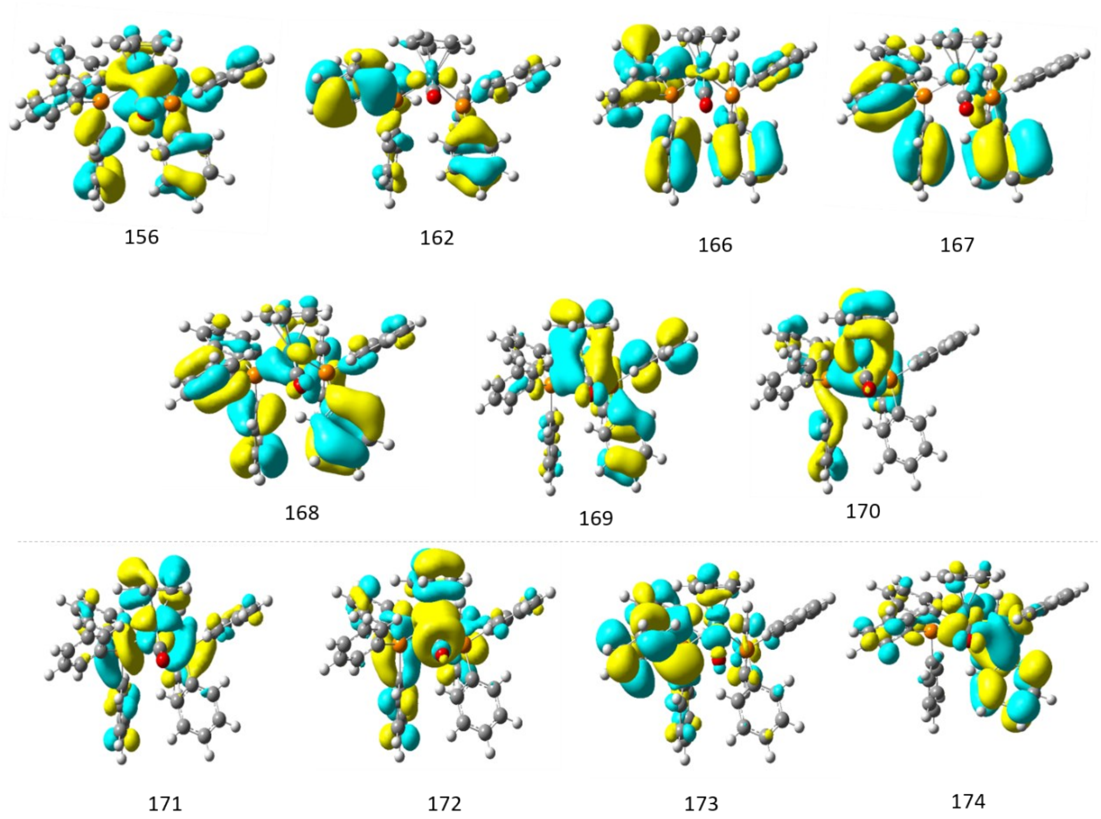

Figure S5: **Molecular orbitals of 1 involved in the transitions predicted by TDDFT calculations between 3.06 – 4.67 eV described in Table S2. The iso-value for each orbital surface is 0.02 e/au^3. The dashed line indicates the se**paration between occupied and unoccupied orbitals.
